# Supplementary material for: The Addition of Hot Water Extract of Juncao-Substrate Ganoderma lucidum Residue to Diets Enhances Growth Performance, Immune Function, and Intestinal Health in Broilers
Source: Animals (Basel). 2024 Oct 11;14(20):2926. doi: 10.3390/ani14202926 (PMC11503797; doi:10.3390/ani14202926)
Supplement: Supplementary file 1 [file animals-14-02926-s001.zip › animals-3204252-supplementary.pdf]

Table S1. Effect of HWE-JGLR on growth performance of 21-day-oldyellow-feather broilers[1]

Items	Groups				*p*–value
	**CON**	**HJ–1**	**HJ–2**	**HJ–3**	
ADFI, g	30.41 ± 1.65	30.42 ± 0.61	30.46 ± 0.78	31.66 ± 1.80	0.300
ADG, g	17.71 ± 0.85	17.81 ± 0.86	17.98 ± 0.34	18.04 ± 0.85	0.863
F/G	1.72 ± 0.04	1.71 ± 0.07	1.69 ± 0.02	1.76 ± 0.07	0.240

[1]ADFI = average daily feed intake; ADG = average daily gain; F/G = feed: gain ratio.
